# A new mechanism of cancer initiation that involves the transformation of hepatocytes into preneoplastic single hepatocytes and minifoci positive for glutathione S‐transferase P‐form (GST‐P) in rat livers: 3D analysis using a vibratome

**DOI:** 10.1002/cam4.70165

**Published:** 2024-09-24

**Authors:** Kimihiko Satoh

**Affiliations:** ^1^ Department of Biomedical Sciences Graduate School of Health Sciences, Hirosaki University Hirosaki Japan; ^2^ Department of Medical Welfare Akita University of Nursing and Welfare Odate Japan

**Keywords:** 3D analysis, cancer initiation, chemical hepatocarcinogenesis, GST‐P, tumor markers, vibratome

## Abstract

**Background:**

Cancer initiation has long been “unknowable” in biology and medicine. In 1987, however, Moore and our research group observed single hepatocytes and minifoci that were strongly positive for glutathione S‐transferase P‐form (GST‐P) in the rat liver as early as 2 to 3 days after initiation by diethylnitrosamine prior to the induction of GST‐P^+^ foci and nodules. The induction of GST‐P^+^ single hepatocytes, precursors of GST‐P^+^ foci and nodules, was considered genetic. But, the details of the induction mechanism have remained unclear despite various examinations over a long period.

**Methods:**

Male Sprague‐Dawley rats (aged 6 weeks) were fed a basal diet containing either benzyl isothiocyanate (BITC, 0.5% by wt) or 2‐acetylaminofluorene (AAF, 0.04%) *ad* *libitum* for appropriate time intervals. All animals were anesthetized and euthanized. The livers obtained were excised, cut into 3‐ to 4‐mm‐thick slices and fixed in cold acetone at 4 °C. The liver specimens were then sliced into 25‐µm‐thick sections in PBS using an automated microtome (Vibratome 1500 Sectioning System, Vibratome Products, NY, USA). Immunocytochemical staining was performed in free solution, and the results were examined via digital light microscopy (Coolscope, Nikon, Tokyo).

**Results:**

3D analysis using a vibratome showed that GST‐P is rapidly excreted into the bile of the liver of animals in response to strong carcinogenic stress caused by promoters or initiators. “Rapid biliary excretion of GST‐P” was widely and commonly observed in all hepatocytes, GST‐P^+^ single hepatocytes, minifoci, foci and nodules under appropriate conditions. Surprisingly, on the basis of these key findings, a new mechanism of cancer initiation involving the transformation of hepatocytes into GST‐P^+^ single hepatocytes and minifoci in animal livers was identified. In addition, the initiation process was determined to be nongenetic because mutation is an invisible rare event.

**Conclusions:**

This short review describes several details about breakthrough findings on cancer initiation in rat livers, the application of 3D analysis to other cancers and the importance in the genetic analysis in malignant diseases.

## Graphical abstract

A new mechanism of cancer initiation that involves the transformation of hepatocytes into preneoplastic single hepatocytes and minifoci positive for GST‐P was identified in the rat liver on the key finding of “rapid excretion of GST‐P into bile” obtained from 3D immunochemistry using a vibratome for the first time. GST‐P represents glutathione S‐transferase P‐form, a specific marker enzyme of preneoplastic foci and nodules inducible in rat livers.

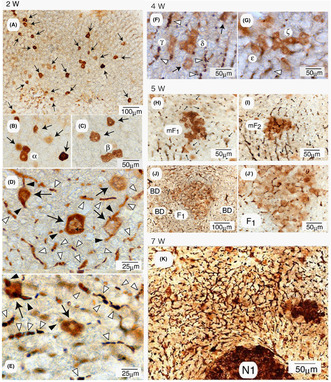

## 1 INTRODUCTION

Whether genetic changes, that is, mutations, are the cause of cancer and the mechanism of initiation remain unclear in biology and medicine. These fundamental issues have been examined solely or exclusively based on the somatic mutation theory (SMT) of Boveri for more than 100 years.^1^ However, Pitot previously noted that mutations might be “unknowable” in principle because the changes are latent and minor.^2^ For example, the probability of detecting mutations is 1 or 2 bases per 2.75 Gbp in rats and 3.1 Gbp in humans, both of which are at the ppb level. Mutation is thus an undetectable or invisible rare event, if not absent. Furthermore, pinpoint analysis of carcinogen binding is markedly more difficult than analysis of mutations in vivo. Prehn questioned whether “cancers beget mutations” or “mutations beget cancers” on the basis of cancer biology data, such as those from a mosaic mouse model.^3^ The SMT hypothesis has also been criticized from various viewpoints.^4^, ^5^ Nonetheless, decisive data are lacking.^6^, ^7^ Although genetic analysis has been the major strategy for gaining insight into the mechanism of cancer initiation, biomarker analysis is also a useful strategy.

In rats with chemical hepatocarcinogenesis, preneoplastic foci and nodules are inducible before neoplastic or malignant lesions are induced. Thus, the induction of foci and nodules has been extensively examined to reveal the mechanism of cancer initiation.^8^, ^9^, ^10^ In 1985, placental glutathione S‐transferase (GST‐P, EC.2.5.1.18) was developed as a specific and sensitive marker enzyme for foci and nodules as well as neoplastic cell populations.^11^, ^12^, ^13^ This marker enzyme has thus far been widely used not only in the basic analysis of chemical carcinogenic processes but also by Ito et al. in medium‐term bioassay, which is an epoch‐making animal test, for initiator and promoter agents as the endpoint marker.^14^, ^15^ In 1987, Moore et al. and our research group detected single hepatocytes and minifoci that are heavily positive for GST‐P prior to the induction of foci and nodules.^16^ Various data indicate that small cell populations could be precursors of foci and nodules. Notably, drastic initial carcinogenic changes that induce GST‐P^+^ single hepatocytes were recently observed in rat livers when analyzed with vibratome‐prepared liver specimens.^17^ GST‐P was found to be rapidly excreted into bile in response to animal exposure to strong carcinogenic stress, and a new mechanism of cancer initiation involving the transformation of hepatocytes into GST‐P^+^ single hepatocytes and minifoci in rat livers was identified.

A biomarker strategy involving 3D immunochemistry using a vibratome was helpful for elucidating the mechanism of cancer initiation at the molecular and cellular levels.

## 2 MATERIALS AND METHODS

### 2.1 Identification of GST‐P^+^ single hepatocytes and minifoci in rat livers

**FIGURE 1:**
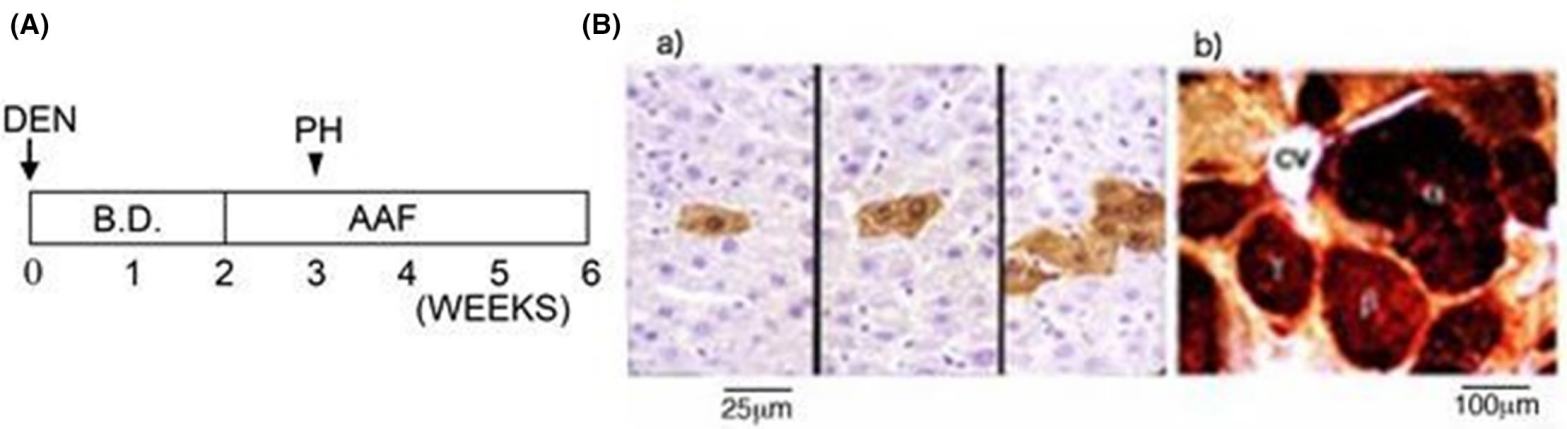
Induction of GST‐P^+^ preneoplastic cell populations in rat livers. (A) Solt–Farber protocol for the induction of preneoplastic cell populations. DEN, injection of diethylnitrosamine in a single i.p. injection at a dose of 200 mg/kg; PH, 2/3 partial hepatectomy; B.D., basal diet; AAF, basal diet containing AAF (0.02%). (B) Immunocytochemical staining patterns of (a) GST‐P^+^ single cells and minifoci induced 2 days after DEN administration and (b) GST‐P^+^ foci and nodules induced after 5 weeks. a), b) were stained with microtome‐ and vibratome‐prepared liver specimens, respectively. CV represents the central vein. Bb was reprinted from Ref. [22] with permission from Wiley.

The rat chemical carcinogenesis protocol described by Solt and Farber, which is shown in Figure 1A, is one of the most efficient and convenient protocols for the induction of preneoplastic foci and nodules in rat livers within a short period of 5–6 weeks.^18^ Diethylnitrosamine (DEN) was used as the initiator carcinogen. After 2 weeks, selection pressure was applied to the animals through the administration of a basal diet containing AAF (0.02%), and 2/3 partial hepatectomy (PH) was performed after 3 weeks. As shown in Figure 1Bb, after 5 weeks, numerous foci and nodules were clearly immunocytochemically observed in the livers of the rats treated with an antibody against GST‐P.^11^, ^12^

**FIGURE 2:**
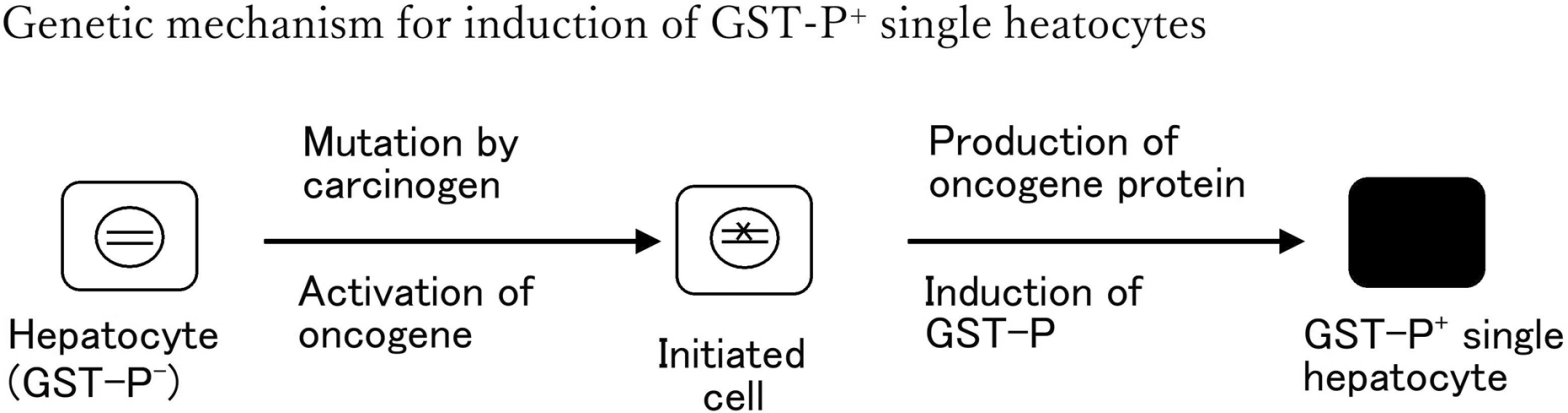
Initiated cell theory for the induction of GST‐P^+^ single hepatocytes according to Farber et al.^8^, ^25^ In this figure, = represents DNA in the nucleus of a hepatocyte, and the mutation is shown by x.

Notably, Moore and our research group observed single hepatocytes and minifoci that were strongly positive for GST‐P (Figure 1Ba) in the rat liver as early as 2–3 days after initiation by DEN prior to the induction of GST‐P^+^ foci and nodules.^16^ These positive cell populations were inducible by many initiator carcinogens but not by promoter agents. Devi and Devaraj reported strong induction of single GST‐P^+^ cells and minifoci in rat livers by cyclophosphamide, an anticancer agent.^19^ Grasl‐Kraupp et al. performed stochastic analysis on the strong induction of positive cells by *N*‐nitrosomorpholine.^20^ However, [^3^T]‐thymidine was no longer incorporated into GST‐P^+^ single hepatocytes and minifoci, which led to suspicion of the genetic induction of cell populations. Furthermore, cell induction kinetics and other data indicated that these positive cell populations were precursors of large foci and nodules.^21^, ^22^, ^23^, ^24^ First, GST‐P^+^ single hepatocytes were regarded as the initiated cells in which the GST‐P gene or some oncogenes were activated by initiator carcinogens, according to the initiated cell theory proposed by Farber et al.,^8^, ^25^ as illustrated in Figure 2. However, when the rats were fed a basal diet containing high concentrations of AAF (0.04%), the frequency of the induction of positive cells per g of liver was 2–3 orders of magnitude greater than the number of mutational changes estimated by Farber et al.^25^ Accordingly, the induction of GST‐P^+^ single hepatocytes was regarded as nongenetic and physiological. Nevertheless, the details of the induction mechanism have remained unclear despite various examinations over a long period.

### 2.2 Detection of drastic initial carcinogenic changes to induce GST‐P^+^ single hepatocytes and minifoci using a vibratome

**FIGURE 3:**
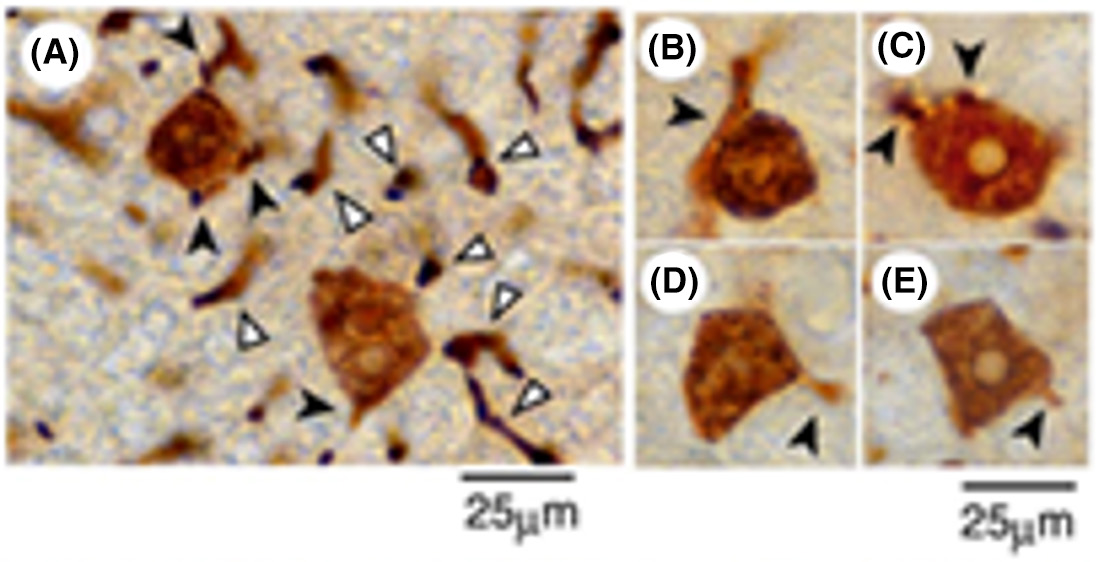
3D analysis of GST‐P^+^ single hepatocytes. (A) Representative staining pattern of GST‐P^+^ single hepatocytes induced in the livers of rats fed a basal diet containing AAF (0.02%) and NAC (0.5%) for 4 weeks. White arrowheads represent staining of canaliculi; (B–E) examples of GST‐P^+^ single hepatocytes positive for the excretory portions (arrowheads). Reprinted from Ref. [17] with permission from Elsevier.

Unexpectedly, the situation has changed almost completely. In contrast to the genetic mechanism of initiated cell theory, prominent initial carcinogenic changes were observed in the livers of animals fed a basal diet containing AAF (0.02%) and NAC (0.5%), followed by immunocytochemical 3D analysis of GST‐P using a vibratome.^17^ As a result, NAC, a radical scavenger, was found to act as a cancer promoter at high concentrations.^26^ Numerous GST‐P^+^ single hepatocytes were observed in the liver after 4 weeks (Figure 3A). Strangely, however, the excretory portions of the GST‐P^+^ single hepatocytes (arrowheads) were also frequently positive for GST‐P (Figure 3B–E). Furthermore, bile canaliculi distant from the positive cells were also heavily but partially stained for GST‐P (Figure 3A, white arrowheads).

### 2.3 Dynamic action of GST‐P to be excreted into bile

**FIGURE 4:**
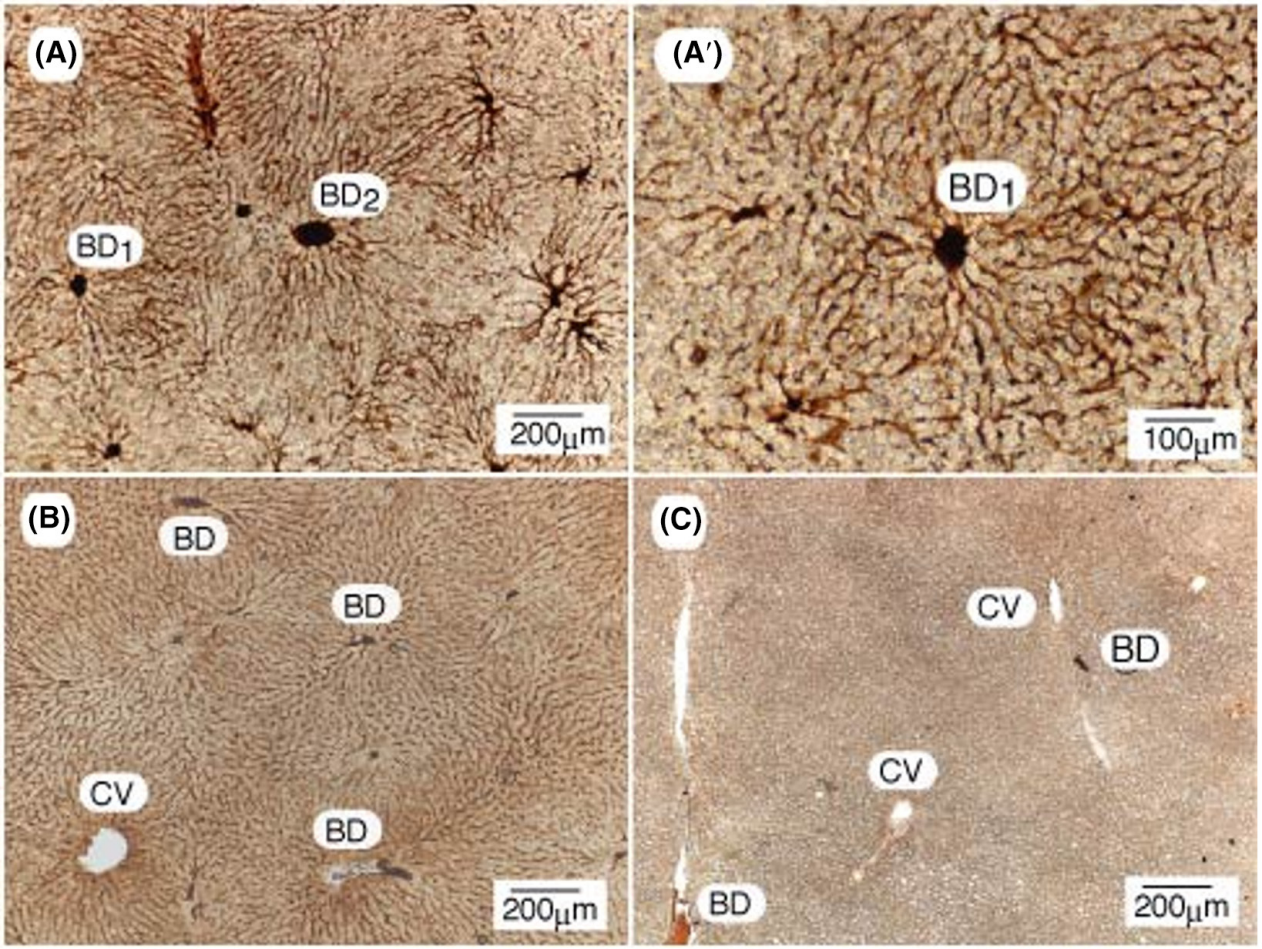
3D analysis of canalicular networks and bile ducts in BITC‐treated rat livers with a GST‐P antibody. (A) and (B) Staining patterns of the livers of rats fed the BITC‐containing diet for 5 and 10 days, respectively. (A') Higher magnification of (A). (C) Staining pattern of the nonfed control. BD, interlobular bile duct; CV, central vein. Reprinted from Ref. [25] with permission from Elsevier.

These questions were then answered as follows. Among the four promoter agents tested, BITC, an anticancer phytochemical,^27^ was noted to be a strong promoter at high concentrations.^26^, ^28^ When the animals were fed a basal diet containing high concentrations of BITC (0.5%) alone, the canaliculi, canalicular networks, and bile ducts showed selective and strong staining for GST‐P after 5 (Figure 4A) and 10 days (Figure 4B), although GST‐P is a cytosolic enzyme. The mean inside diameter of the capillary tubes greatly increased to 6.7 ± 0.5 μm (mean ± SE, *n* = 25). After 10 days, however, the canalicular tubes markedly shrank to 2.6 ± 0.02 μm (*n* = 20), which was still much greater than that of nonfed animals (0.5–1 μm). No staining was observed in the nonfed control group (Figure 4C). The network structure intrinsic to the bile canaliculi was markedly clearer at higher magnification (Figure 4A'). The mean inside diameter of the capillary tubes of the BITC‐treated animals was therefore 5‐ to 10‐fold greater than that of the nonfed controls. Notably, inside the large interlobular bile ducts, strong staining for BD1, BD2, and others was detected. Thus, the canaliculi and bile ducts were no longer stained for GST‐P, but the bile liquid in the biliary tracts was strongly and homogeneously stained for the marker protein. Specifically, although GST‐P is a cytosolic enzyme, its expression in the cytosol of hepatocytes was negative according to immunocytochemistry. Accordingly, these results unambiguously indicate that GST‐P is synthesized rapidly in all hepatocytes of the liver but is excreted rapidly into bile via an unknown mechanism. Rapid biliary excretion of GST‐P is key for determining the mechanism of initiation. Once the dynamic action of GST‐P was revealed, many questions could be easily answered.

### 2.4 3D analysis of preneoplastic cells induced in the rat liver by AAF

**FIGURE 5:**
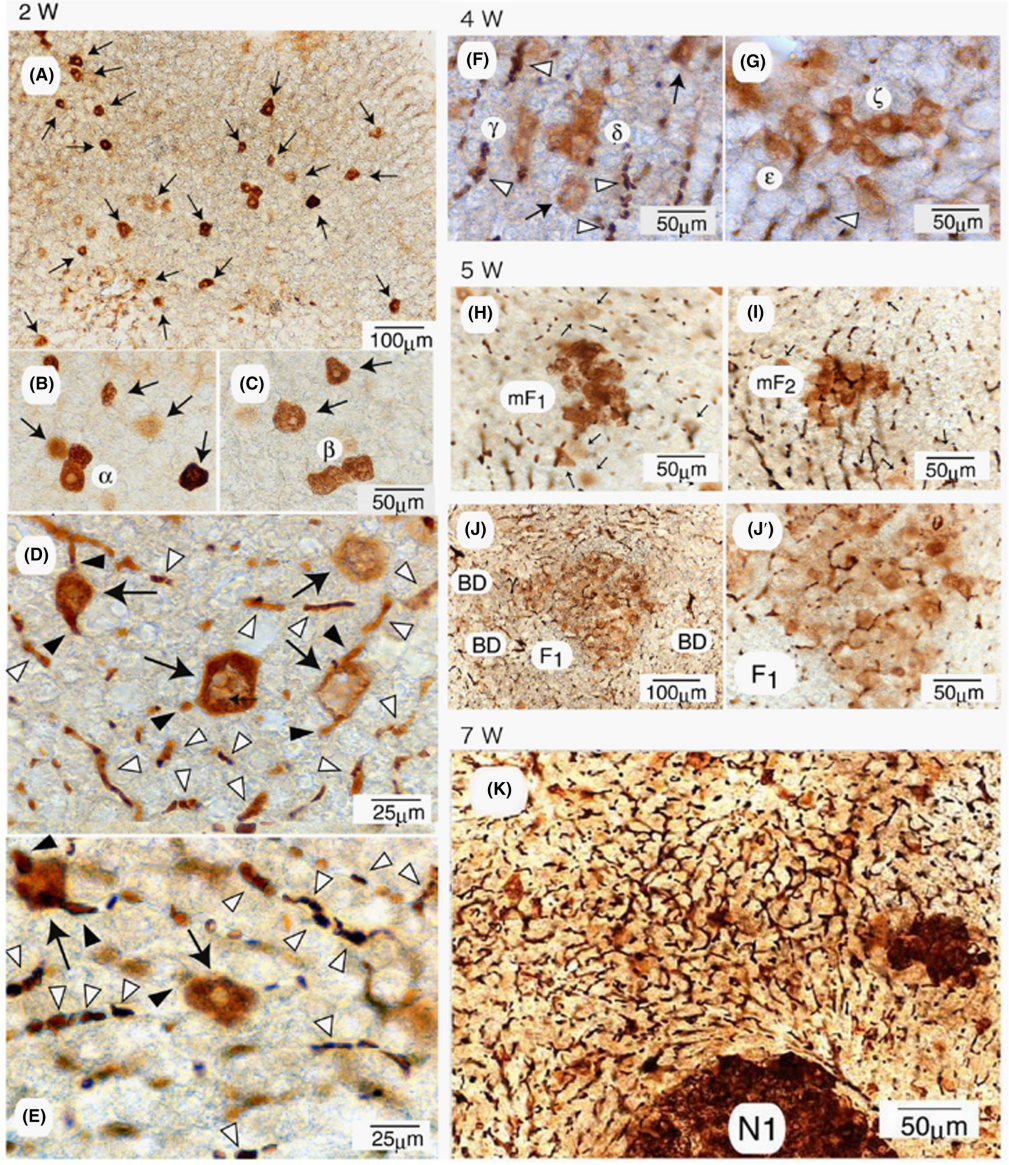
3D analysis of preneoplastic cell populations induced in rat livers by AAF. The animals were given dietary AAF (0.04%) for 7 weeks. (A–E), Single hepatocytes and minifoci induced after 2 weeks; (F, G), minifoci induced after 4 weeks; (H–J), minifoci and foci induced after 5 weeks; (K), a nodule induced after 7 weeks. Arrows, GST‐P^+^ single hepatocytes; arrowheads, canaliculi linked to GST‐P^+^ single cells; white arrowheads, GST‐P^+^ canaliculi. Reprinted from Ref. [25] with permission from Elsevier.

The time course for the induction of preneoplastic cell populations in the rat liver was examined by the administration of a basal diet containing high concentrations of AAF (0.04%) for 7 weeks.^29^ As a result, biliary excretion of GST‐P was widely and commonly observed in all hepatocytes, GST‐P^+^ single hepatocytes, minifoci, foci, and nodules, although to varying degrees. Figure 5 shows representative images of their induction in the livers of the animals. After 2 weeks, numerous GST‐P^+^ single hepatocytes were detected, as shown in Figure 5A (arrows). A few minifoci are also shown in Figure 5B,C, in which minifoci α and β were composed of 2 and 3 positive cells, respectively. Although partial but prominent biliary excretion of GST‐P was detected for single hepatocytes, as shown in Figure 5D,E and revealed by staining of the excretory portions of GST‐P^+^ single hepatocytes (black arrowheads), canaliculi distant from single hepatocytes were also positive for GST‐P (white arrowheads). The mean inside diameter of the canaliculi was as large as 6.9 ± 0.4 μm after 2 weeks, which was markedly greater than that found in the livers of nonfed animals (0.5–2 μm). GST‐P^+^ precipitates were detectable within the enlarged tubes (white arrowheads), indicating that GST‐P accumulated at high concentrations in the capillary tubes. After 4 weeks, many GST‐P^+^ minifoci were induced (Figure 5F,G), and minifoci γ, δ, ε, and ζ were composed of 3, 6, 4 and 7 positive cells, respectively. These cells are tightly linked to each other via canaliculi, and the inside diameter of the GST‐P^+^ canaliculi was as large as 8.1 ± 0.9 μm. Figure 5H,I show two representative minifoci, mF1 and mF2, which are composed of approximately 18 and 15 GST‐P^+^ cells, respectively. Figure 5J,J' show a focus (F1) and a high‐magnification image. Biliary excretion was observed in the outer regions, as well as inside of these minifoci (mF1 and mF2) and the focus (F1). After 7 weeks, fine and dense GST‐P^+^ canalicular networks were most prominent in the central nonnodular regions, as shown in Figure 5K, where N1 represents a nodule. The mean inner diameter of canaliculi in the nonnodular regions was 3.3 ± 0.2 μm. Overall, the hepatocytes in the canalicular networks were all negative for the marker enzyme, but the canaliculi were partially or completely positive.

Taken together, these findings indicate that GST‐P is induced rapidly in all hepatocytes but was excreted rapidly into bile in animals in response to strong carcinogenic stress. Prominent biliary excretion of GST‐P was also observed with bile duct epithelial cells.^17^ For more details, see references 17 and 25.

## 3 DISCUSSION

### 3.1 Biochemical characteristics of GST‐P and its new function: Rapid biliary excretion of GST‐P

The biochemical and physiological characteristics of GST‐P are as follows. Glutathione S‐transferase (GST) is an antioxidant enzyme that, together with superoxide dismutase, epoxide hydrolase, catalase, glutathione peroxidase and others, acts as a radical scavenger under conditions associated with oxidative stress.^13^, ^30^ GSTs catalyze the conjugation of GSH with numerous exogenous and endogenous molecules, including carcinogens, as well as binding protein of carcinogens. Among the three major cytosolic GSTs of the Alpha, Mu, and Pi classes of mammalian species, rat GST‐P belongs to the Pi class of GSTs. The substrate specificity of GST‐P is very broad, but it is fairly selective for ethacrynic acid, acrolein, BITC, and others.^13^, ^31^, ^32^ GST‐P protein expression is very low in normal rat livers but very high in the livers of rats bearing foci and nodules.^11^, ^13^ Muramatsu et al. revealed that the GST‐P gene contains silencing elements, GPSs 1, 2 and 3, as well as antioxidant responsive elements ARE/TRE and enhancer GPE1 elements in the 5'‐upstream regions.^33^, ^34^, ^35^ These enhancer elements could thus respond to the increase in endogenous carcinogens generated from lipid peroxidation. These data therefore indicate that GST‐P expression is significantly suppressed under normal conditions but strongly activated in response to the administration of exogenous carcinogens to animals.

In addition to the extraordinarily high specificity and sensitivity of GST‐P as a marker enzyme for preneoplastic cell populations,^11^, ^12^, ^13^ the marker enzyme is excreted rapidly into bile in aminals in response to strong carcinogenic stress either with AAF, an initiator, or BITC, a promoter agent.^29^ Once the new function was known, the mechanism of GST‐P^+^ single hepatocyte and minifocus induction was unambiguously solved as follows.

### 3.2 A new mechanism of cancer initiation that involves the transformation of hepatocytes into GST‐P^+^ single hepatocytes and minifoci in the rat liver

**FIGURE 6:**
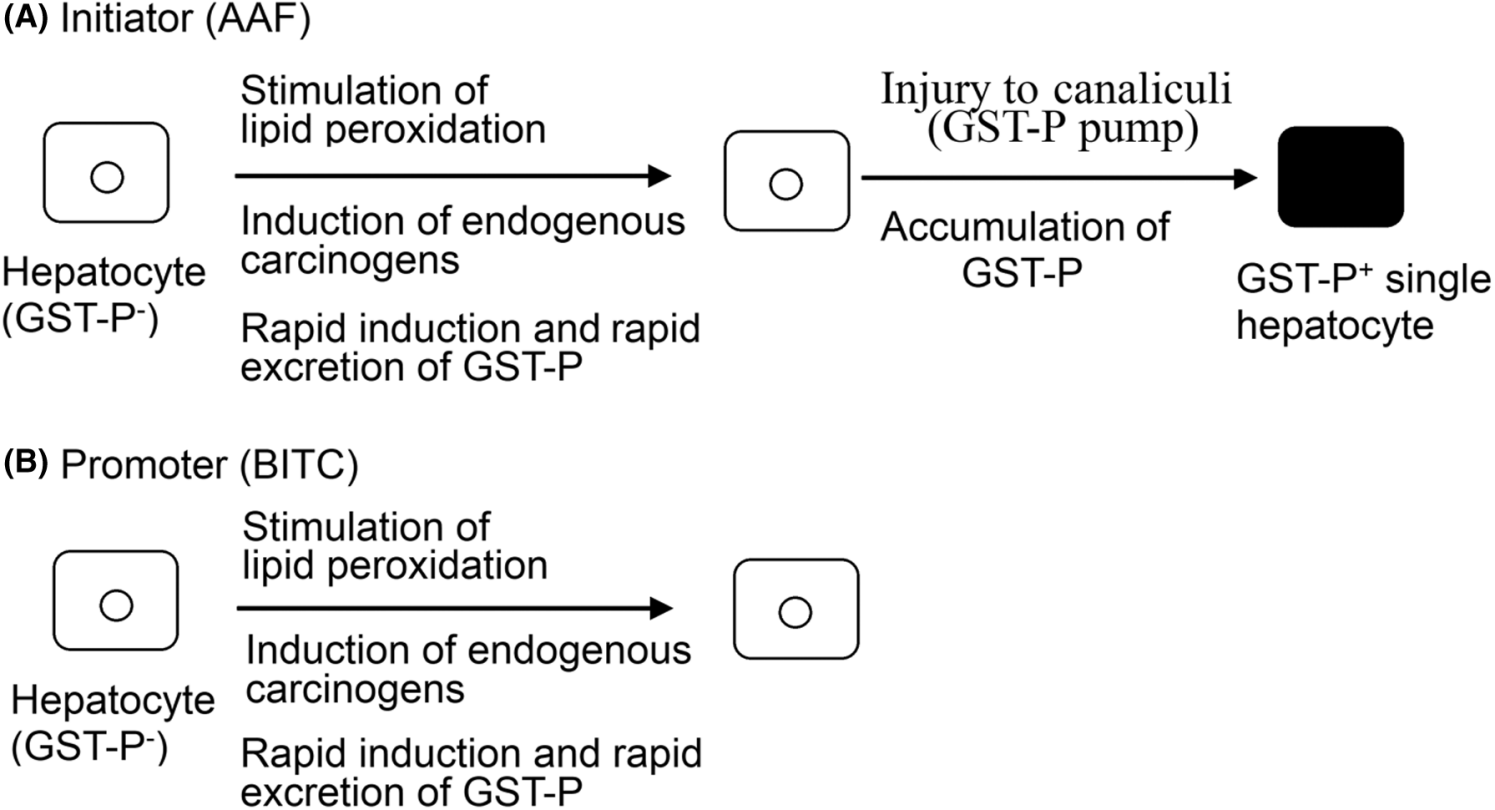
Carcinogenic action of initiator and promoter agents on hepatocytes, which give rise to single GST‐P^+^ hepatocytes and intact hepatocytes, respectively, in rat livers.

On the basis of the findings on the “rapid biliary excretion of GST‐P”, a new mechanism for the induction of GST‐P^+^ single hepatocytes is illustrated in Figure 6A,B as a working hypothesis. In the normal rat liver, endogenous carcinogens, such as acrolein, malondialdehyde, and 4‐hydroxynonenal, are constitutively generated intracellularly, although at very low concentrations^36^, ^37^, ^38^; thus, GST‐P protein expression is very low.^11^ When animals are subjected to strong carcinogenic stress, both the initiator and promoter act on all hepatocytes to stimulate lipid peroxidation and thus generate endogenous carcinogens.^39^, ^40^, ^41^ GST‐P is then induced to detoxify these toxicants. Alpha and Mu class GSTs, as well as other detoxifying enzymes, are also induced but are irrelevant to the phenotypic conversion of hepatocytes. Kinetically, both the rate of intracellular induction of GST‐P and the rate of excretion are very rapid, and GST‐P is immunocytochemically undetectable in hepatocytes. The initiator then injures the canaliculi/GST‐P pump to decrease the rate of excretion of GST‐P into bile, resulting in the intracellular accumulation of GST‐P, which gives rise to GST‐P^+^ single hepatocytes, as shown in Figure 6A. Minifoci are then formed through the penetration of carcinogens and GST‐P, as shown in the proposed initiation model; in this model, the GST‐P pump was postulated for excretion of the marker protein because canalicular networks showed uniform staining for GST‐P in animals treated with BITC alone.^29^ GST‐P is also induced in hepatocytes by promoter agents, but this induction does not give rise to GST‐P^+^ single hepatocytes, as shown in Figure 6B.

As a result, mechanism A represents the trick of initiation that induces GST‐P^+^ single hepatocytes and minifoci in the rat liver. This new mechanism is entirely different from the initiated cell theory shown in Figure 2. Mechanism B is also a new mechanism for detoxifying not only promoter agents but also initiator agents. Therefore, it is reasonable that cells and animals eliminate toxic compounds as soon as possible following exposure to carcinogens. Two mechanisms, A and B, account for the various problems associated with the induction of preneoplastic cell populations. To our knowledge, this study is the first to report the molecular and cellular mechanism of cancer initiation, which was revealed through experimental examinations of rat livers in vivo. Although further examination is needed, the fact that these nongenetic mechanisms can be investigated by ordinary biochemical techniques is highly advantageous because genetic mechanisms are experimentally unexaminable in principle.^2^, ^6^, ^7^

### 3.3 Counterevidence against the genetic mechanism of initiation

Notably, the induction of GST‐P^+^ single hepatocytes and minifoci shown in Figure 6A was determined to be nongenetic in nature. Mutation is a rare event that occurs at the ppm level or less in the somatic cells of experimental animals and humans.^2^, ^25^, ^42^, ^43^ Mutations are therefore undetectable or invisible in practice if not absent, as summarized in Table 1. Thus, experimental detection itself provides strong evidence that disproves the genetic mechanism of initiation. Quantitatively, as many as 517,000 ± 86,000 GST‐P^+^ single hepatocytes were induced per g of liver in animals fed a basal diet containing AAF (0.04%) for 4 weeks when examined stereologically.^24^

**TABLE 1:** Comparison of nongenetic and genetic changes that occur in the somatic cells of experimental animals and humans.

	Nongenetic changes	Genetic changes/mutations^a^
Reaction	Biochemical, physiological	DNA base modification,^b^ mutation
Frequency	High to low (%)	Very low, minor, latent, rare (ppm to ppb)
Experimental detection	Positive, visible	Negative, invisible

^a^ Refs. [2, 26, 43, 44].

^b^ Pin‐point binding of carcinogens to the causal base(s) of mutation.

In accordance with this counterevidence, mechanism A in Figure 6 is nongenetic because initiator carcinogens act to stimulate lipid peroxidation followed by injury to bile canaliculi/GST‐P pumps, giving rise to GST‐P^+^ single hepatocytes and minifoci. In fact, their induction is acute and drastic.^16^, ^17^, ^19^, ^20^, ^21^, ^22^, ^23^, ^24^, ^29^ Thus, rare events cannot cause these changes in the animal liver within a short period of 2–3 days of DEN treatment or within approximately 1 week of AAF treatment. In addition, this rare event might be totally insufficient or inefficient at eliminating carcinogens from somatic cells in animals exposed to carcinogens. Considering these experimental findings and evidence, the mechanism through which cancer initiates the induction of GST‐P^+^ single hepatocytes and minifoci can be determined to be nongenetic in nature. Importantly, the initiation process is determinable to be nongenetic. Cancers are considered “incurable” diseases since genetic changes, that is, mutations, might be irreversible.^1^, ^6^, ^7^ In contrast, nongenetic changes are reversible in general, which may mean that cancers are “curable” in nature. There are many contradictions to the genetic mechanism, which will soon be reported elsewhere.

### 3.4 Ex vivo and in vivo expression of GST‐P in the absence of exogenous carcinogens

For example, mechanism A accounts well for the following facts. Curiously, GST‐P is strongly expressed in primary cultures of hepatocytes as well as in liver organoids from rats and humans without the addition of exogenous carcinogens.^44^, ^45^, ^46^, ^47^ These findings may be due to the severe damage caused by canaliculi/GST‐P excretory pumps in hepatocytes during the isolation of hepatocytes from liver tissue through perfusion and collagenase treatment.^48^, ^49^ Although endogenous carcinogens generated under ex vivo conditions might be present at very low concentrations, these toxicants accumulate gradually to substantially high concentrations intracellularly under culture conditions, resulting in the induction of GST‐P and other detoxifying enzymes. Aoki et al. reported specific induction of GST‐P in primary cultured rat liver parenchymal cells by coplanar polychlorinated biphenyl congeners.^50^ However, considering the “biliary excretion of GST‐P into bile,” the amount of GST‐P protein induced in the hepatocyte culture may be much greater than that detected by 2D gel electrophoresis.

Various cell lines, such as dRLh cells derived from rat hepatoma, F9 cells from mouse embryonal carcinoma and HeLa cells from human uterus carcinoma, are known to express GST‐P at substantially high levels.^39^ Most of the ascites‐derived hepatomas of rats generally exhibit low GST‐P expression.^11^ Thus, the level of Pi class GST expression may depend on the balance of intracellular induction and the efficiency of the excretory pump. The spontaneous induction of GST‐P^+^ single hepatocytes, especially in normal F‐344 rat livers, is also notable.^12^, ^14^ As described above, this process occurs upon inactivation of the excretory pump by various endogenous and exogenous factors in vivo. In contrast, the frequency of specific activation of the GST‐P gene by spontaneous mutation is extremely low, with a frequency of 0. In addition to the low frequency of mutation itself, spontaneous mutation must occur specifically in particular base(s) of the GST‐P gene (2.778 bp in the structural gene and 2.5 kb in the regulatory gene).^33^, ^34^ In addition, mutated base(s) may be repaired readily by DNA polymerases.

## 4 APPLICATION OF THE NEW METHODOLOGY TO OTHER CANCERS

Notably, hamster buccal pouch carcinogenesis is very similar to chemical hepatocarcinogenesis in rats, as described above.^51^, ^52^ It is clear that GST‐P is excreted from epithelial cells because strong staining of the pathways was observed even through examination with microtome‐prepared tissue specimens after topical application of the DMBA (7,12‐dimethylbenz[α]anthracene) initiator followed by the TPA promoter to the animals. Therefore, according to the mechanisms shown in Figure 6, GST‐P may be inducible intracellularly but excreted rapidly from cells upon application of either DMBA alone or TPA alone.

In Long‐Evans Cinnamon rat carcinogenesis, the causal gene has been identified to be deletion of the copper transporting ATPase gene, Atp7b, which is homologous to the Wilson's disease gene of humans.^53^, ^54^ These animals are known to be susceptible to DEN and AAF, which can induce numerous GST‐P^+^ preneoplastic cell populations in the liver according to the Solt–Farber protocol.^55^ In spontaneous carcinogenesis, Cu ions may therefore act as weak promoters, and bilirubin metabolites may act as weak initiators to induce GST‐P^+^ cell populations in the animal liver.

Pi class GSTs are known to be closely associated with the carcinogenic processes of various cancers in humans.^13^, ^40^, ^41^ These new findings and methodologies may be helpful for elucidating the initial carcinogenic processes of a wide variety of malignant diseases at the molecular and cellular levels. These biochemical data may also be valuable for genetic analysis of carcinogenic processes.^56^, ^57^, ^58^, ^59^

## 5 CONCLUSIONS

Cancer initiation was found to be examinable in rats with chemical hepatocarcinogenesis because drastic initial carcinogenic changes that induce GST‐P^+^ single hepatocytes and minifoci, precursors of foci and nodules, were detected in rat livers. 3D analysis using a vibratome revealed that GST‐P is rapidly synthesized in all hepatocytes but is rapidly excreted into bile in response to the strong carcinogenic stress applied to the animals. On the basis of these key findings, a new mechanism of cancer initiation to transform GST‐P^−^ hepatocytes into GST‐P^+^ single hepatocytes and minifoci in the animal liver was identified, albeit tentatively. Importantly, initiation was determined to be nongenetic because mutation is an invisible rare event. This new methodology may be helpful for gaining further insight into the mechanism of cancer initiation in other cancers at the molecular and cellular levels. The earliest or initial carcinogenic changes, that is, cancer initiation, may also be of significance in genetic analyses, such as the adenoma‐carcinoma sequence, oncogene activation, driver mutations, and signal transduction.

## 6 ADDENDUM: THE SMT HYPOTHESIS IS AN EXISTENCE PROBLEM IN BIOLOGY AND MEDICINE

The so‐called existence problem is known well for the intrinsic difficulties of proving the nonexistence of solution(s) in mathematics. One famous existence problem is Felmat's Last Theorem, which took 360 years to solve; this problem was solved recently by Prof. Andrew Wiles.^60^ There is a high risk in solving the mechanism of cancer initiation, that is, the cause or origin of cancer in experimental animals or humans, according to the SMT hypothesis of Boveri.^1^ This hypothesis has been widely or exclusively applied to elucidate mechanisms such as the initiated cell theory and two‐step or multistep carcinogenesis theories in cancer research.^61^, ^62^ In fact, there are two cases for this. One is the case in which the SMT is correct, and the mechanism of cancer initiation may be solved or elucidated sooner or later owing to the rapid progress in modern gene technologies. The other is the case in which SMT is incorrect. In this case, trying to find nonexistent genetic changes, that is, mutations, is endless and futile work. The newly revealed mechanism of initiation in rat livers in vivo but corresponds to the latter case.

## AUTHOR CONTRIBUTIONS

**Kimihiko Satoh:** Conceptualization (lead); data curation (lead); formal analysis (lead); funding acquisition (lead); investigation (lead); methodology (lead); project administration (lead); resources (lead); software (lead); supervision (lead); validation (lead); visualization (lead); writing – original draft (lead).

## FUNDING INFORMATION

This study was supported in part by the Hirosaki University Fund for Promotion of International Scientific Research (Hirosaki, Japan) and Grants‐in‐Aid for Scientific Research from the Ministry of Education, Culture, Sports, Science and Technology, Japan (grant numbers: 60570104 and 07457579).

## CONFLICT OF INTEREST STATEMENT

The author declares no conflicts of interest to disclose.

## Data Availability

All the data needed to evaluate the conclusions in the paper are presented in the paper and in the references cited in this article.
